# Hospital Food Service Experiences Between Older Patients From English‐ and Non‐English Speaking Backgrounds in a Large Public Hospital in Australia: A Qualitative Analysis

**DOI:** 10.1111/hex.70444

**Published:** 2025-09-26

**Authors:** Caitlin Wyman, Morgan Pankhurst, Sue Yi Lee, Shahid Ullah, Edwin C. K. Tan, Bich Tran, Yogesh Sharma, Zhaoli Dai

**Affiliations:** ^1^ College of Medicine and Public Health Flinders University Adelaide Australia; ^2^ College of Nursing and Health Sciences Flinders University Adelaide Australia; ^3^ School of Pharmacy The University of Sydney Sydney Australia; ^4^ Bureau of Health Information, NSW Health Sydney Australia; ^5^ Department of Acute and General Medicine Flinders Medical Centre Adelaide Australia; ^6^ School of Population Health, Faculty of Medicine and Health University of New South Wales Sydney Australia; ^7^ Ageing Futures Institute, Faculty of Medicine and Health University of New South Wales Sydney Australia

**Keywords:** cultural diversity, hospital food service, lived experiences, nutrition, patient satisfaction

## Abstract

**Introduction:**

With Australia's ageing population increasing and the fast‐growing number of migrants from culturally diverse backgrounds, ensuring the quality of care, including hospital food services, is critical. Meals tailored to patients' needs have been shown to reduce complications and lower hospital costs, making the quality of hospital food service a key factor in overall patient satisfaction. However, data on the lived experiences of older patients from non‐English‐speaking backgrounds (NESBs) regarding hospital food services and meal quality remain limited.

**Methods:**

Semi‐structured interviews were conducted with patients aged 65 years or older from Australian Anglo and NESBs to explore their experiences with hospital food services. A reflexive thematic analysis was undertaken, with the identified similarities and differences between the two groups to inform the development of themes.

**Results:**

The study included 15 Australian‐Anglo background patients (mean age: 83) and 15 NESB patients (mean age: 78). The interviews revealed that care priorities, cultural identity and health needs shaped patients' experiences of hospital food, with four themes being identified: (1) No Complaint Mindset; (2) Food and Cultural Identity; (3) Experiences of Food Service and (4) Nutrition and Health. Through these themes, we found that both groups shared a ‘no complaints’ mentality, with mixed experiences of hospital meals, and preferred smaller meals. NESB participants described limited cultural inclusivity in hospital food service as being related to the lost connection between food and their cultural and linguistic backgrounds. In the same group, English language barriers hinder communication with food service staff to meet dietary needs.

**Conclusion:**

The findings from our qualitative interviews suggest that hospital food services may consider offering culturally familiar options to accommodate patients from diverse cultural and linguistic backgrounds and fostering open and effective communication regarding patients' meal preferences and dietary needs, especially for those with limited English proficiency.

**Patient or Public Contribution:**

The interview guide and process were developed based on feedback from clinicians at Flinders Medical Centre. The study findings and report were reviewed by an experienced consumer representative and a dietetic department head in an independent hospital. Both critically reviewed the manuscript, interpreted the results and contributed revisions based on their lived experiences and clinical expertise. As a result, the final manuscript is a collaborative effort between researchers and public stakeholders, including patient representatives and service providers.

## 1 Introduction

Australia's ageing population is increasing, with the fastest projected growth among migrants aged 65 years and above from diverse ethnic and cultural backgrounds, particularly those originating from Asia ( > 200% growth), the Middle East ( > 150% growth) and Sub‐Saharan Africa ( > 200% growth) [1]. These demographic changes necessitate the development of tailored health policies and service delivery to address the unique needs of this diverse population. For example, poor access to quality healthcare is evident in previous studies [2, 3], including a systematic review [3], where cultural differences in values, beliefs and communications are suggested as the primary cause of addressing inclusion and equity in health services. One area in which to improve inclusion and equity in the healthcare system is food quality in hospital meals. To many racial and ethnic minority patients, hospital food is a salient reminder of the quality of care.

In hospital settings, the quality of food service plays a significant role in patient satisfaction with overall hospital care [4, 5, 6, 7, 8, 9] and in reducing the risks of developing complications and prolonging the length of hospital stay [10]. This can be explained by dissatisfaction with food service affecting food intake, which, in turn, increases the risk of malnutrition [11, 12]. By contrast, hospital meals tailored to meet patients' clinical needs result in cost benefits and effectiveness, with 20%–30% lower complications, 25%–50% dialysis rates and hospitalisation costs than patients on regular hospital meals [10]. This evidence suggests that hospital food service plays a vital role in patients' health and recovery. Indeed, the National Safety and Quality Health Service Standards indictate that health service organisations must provide comprehensive care to identify patients' needs and minimise patient harm, including ensuring their nutritional needs and requirements are met [13].

Our previous analysis, using a large population‐based survey of 16,919 public hospital patients in New South Wales, Australia, found that hospital food ratings were significantly associated with higher odds of dissatisfaction and higher odds of developing complications and experiencing delayed discharge. Concerningly, in patients from non‐English‐speaking backgrounds (NESBs), the magnitude was 10‐fold higher for care dissatisfaction and more than three times the odds of delayed discharge [14]. Studies from other countries in clinical settings corroborate this observation [15, 16]. For example, in a US study of 229 low‐income patients of non‐Hispanic white and African American adults, a sense of control and healthcare providers' cultural sensitivity played a significant role in the dietary adherence and satisfaction of African American patients [15]. Providers' cultural sensitivity had a significant positive indirect effect on patient care satisfaction, with a larger effect size for white Americans than African Americans, particularly in terms of trust in the provider, care satisfaction and patient interpersonal control. Additionally, an Australian study found that a survey of 83 aged care residents from NESBs were over threefold satisfied with the care facility due to the presence of bilingual staff members [6]. Conversely, when racial and ethnic minority patients were perceived as a homogenous group, they developed a distrust of the preparation and discontent of the hospital food served, leading to insufficient nutritional intake reported in a qualitative study [16].

However, there is a lack of data to understand the lived experience of hospital food service and meal quality among older patients from culturally diverse and NESBs. Therefore, this study addressed this gap by examining the experiences and preferences of older hospital patients regarding food service and nutrition, identifying enablers and barriers, and assessing whether and how cultural and linguistic backgrounds influence food consumption in hospital meals.

## 2 Methods

### 2.1 Study Design

A phenomenological perspective was employed using qualitative methodology to explore and describe participants' subjective experiences [17] of the hospital food service. Our research design, involving interviews with English and NESB older adults, was informed by the quantitative findings from patient surveys across 75 public hospitals [14]. The term NESB is commonly used in Australia to refer to individuals born in non‐English‐speaking countries, highlighting the risks associated with English fluency and reinforcing the barriers faced by this group in society. In this study, we defined people from NESB as those who were not born in Australia and whose first language was not English. The two research assistants, one with a dietetic degree (who had worked on research in nursing homes and with multicultural backgrounds) and the other as a postgraduate medical student (from a non‐Anglo culture), were trained before conducting the interviews and had previous experience in conversations with older adults from English and NESBs. Our interview prompts focused on understanding participants' experiences in consideration of the social and cultural contexts of the hospital food service, as such, giving voice to health and social disparities [14].

This study adheres to the Consolidated Criteria for Reporting Qualitative Research (COREQ) guidelines (Supplementary File 1) [18].

### 2.2 Participants and Recruitment

Convenience sampling was used to recruit English and NESB older adults aged 65 years and above, defined by the Australian Institute of Health and Welfare [19], from the Department of General Medicine long‐stay unit at Flinders Medical Centre. Participants admitted with a highly contagious infectious disease (e.g., Covid‐19), requiring palliative care, on parenteral nutrition, enteral nutrition, or nil per os (nil‐by‐mouth) orders were excluded. These criteria ensured capturing hospital food service in a standard setting rather than catering to clinical needs, which require clinical speciality in food or nutrient preparations. Patients who were identified as eligible for the study were approached by the physician after screening. A total of 30 participants were recruited from May 2023 to October 2024. This sample size was identified as appropriate based on a previous systematic review [20], which indicates empirical data reached saturation between 9 and 17 interviews. Informed written consent was obtained from participants before all interviews. Before the written consent was provided to the patient in English, the resident registrar had already communicated with them to obtain verbal consent. Additionally, patients from NESBs had their family members accompany them when they returned it. The research team had no prior relationships with participants.

### 2.3 Setting

The hospital food service requires patients to verbally nominate their main meal (breakfast, lunch and dinner) orders one day in advance, with their choices electronically recorded by nursing staff. The menu system offers patients two to three options per meal. Meal trays are delivered to patients' overbed tables during designated mealtimes and collected by patient services assistants. Morning and afternoon tea trolleys are also available to patients between main meals.

### 2.4 Data Collection

The first and third authors conducted separate in‐person interviews at the patient's bedside, between mealtimes, using a semi‐structured interview guide (Supplementary File 2). The first author (Anglo‐Australian) conducted interviews with one NESB individual and one Anglo‐Australian individual, and the third author (non‐Anglo‐Australian) conducted interviews with 14 NESB individuals. Other Anglo‐Australian interviews were conducted by the previous research assistant, who spoke fluent English and was not involved in the preparation of this paper. Each of them conducted the interviews separately rather than jointly. All interviewers received the same standardised training before conducting the interviews to reduce biases and inconsistencies. All interviewers were female research assistants. To maintain anonymity, English‐speaking participants were assigned to Group A, and NESB participants were assigned to Group B, with sequential numeric codes assigned to each participant. Demographic information was collected on age, gender, highest level of education, birth country, first language, diagnosis and length of stay. All interviews were audio‐recorded with the participants' and their families' (if present) permission and transcribed verbatim by the research team, along with field notes being documented. Professional translation services via telephone were available to NESB participants upon request.

### 2.5 Data Analysis

Interview transcripts were analysed using Braun and Clarke's six‐phase approach to thematic analysis [21] and documented using NVivo software. Phase 1 involved familiarisation with the dataset by listening to the recordings and reading the transcripts [21]. In the second phase, the data were coded by one researcher using a combination of deductive, inductive and descriptive/in vivo coding [21]. In Phase 3, similar codes were grouped in the English and NESB datasets and then developed into separate categories and themes through iterative discussions with the research team [21] among the first, second, third and senior authors, as well as the resident registrar, who was acknowledged in this paper. The preliminary themes were further refined in Phase 4, with similarities and differences identified between the two groups before the themes were reviewed, named and defined in Phases 5 and 6 [21]. Member checking was not conducted due to resource constraints.

## 3 Reflexivity Statement

As nutrition and clinical researchers, we developed the semi‐structured interview guide drawing on both our clinical experience with older patients and existing literature on hospital food services. Throughout data collection and analysis, we were mindful of our positionality and sought to minimise bias. During interviews, we focused on listening closely to patients' accounts. In summarising, thematising and interpreting the data, we aimed to remain neutral, setting aside our own assumptions and privileging the voices and perspectives of the participants.

## 4 Results

### 4.1 Patient Demographics

A total of 30 interviews, 15 individuals per group, were conducted between November 2023 and October 2024. In the English‐speaking group, participants had a median age of 83 years (range 72–91); most were male (*n* = 11) and had an education level below high school (*n* = 11). In the NESB group, participants had a median age of 78 years (range 73–85), with most being female (*n* = 9) and having a high school education or higher (*n* = 7). The majority of the NESB participants were born in Europe (*n* = 10), with one‐third (*n* = 5) utilising translation services during their interviews. The length of stay for participants before their interviews was similar across both groups, with most (26/30) having been admitted to the hospital for less than 1 week. In both groups, the most common reason for hospitalisation was due to musculoskeletal conditions, followed by cardiovascular and respiratory diseases. Table 1 summarises the participants' demographic characteristics, with further details provided for each participant in Supplementary File 3.

**Table 1:** Demographic and patient characteristics in the study.

Patient characteristics		
	English‐speaking (*n* = 15)	Non‐English‐speaking background (*n* = 15)
Age, years [median (range)]	83 (66–93)	78 (68–92)
Sex [*n* (%)]		
Male	11 (73.3)	6 (40.0)
Female	4 (26.7)	9 (60.0)
Level of education (*n*)		
Below High School	11	5
High School	2	7
University	2	3
Use of translation services (*n*)		
Yes	0	5
No	15	10
Length of stay, days [median (range)]	7 (3–70)	7 (3–28)
Interview length, minutes [median (range)]	23 (16–31)	12 (7–28)

### 4.2 Themes

Thematic analysis of the interview transcripts identified four key themes that reflect the experiences of older patients with hospital food services. These themes were: (1) No Complaints Mindset including feelings of acceptance and satisfaction; (2) Food and Cultural Identity encompassing sub‐themes of assimilation, lack of cultural inclusivity and acceptance of institutional norms; (3) Experiences of Food Service wherein participants discussed food quality, food presentation, menu variety and staff interactions; and (4) Nutrition and Health describing the impact of illness on appetite and food intake. A summary of all themes is provided in Table 2, with further details below.

**Table 2:** Summary of themes with representative quotations from English and non‐English‐speaking participants.

Theme/Sub‐theme		
1. No Complaints Mindset: Patients voiced a shared mentality of not wanting to complain about the food service, believing it was good enough, particularly considering the complexities of the large hospital system. It was evident that the non‐English‐speaking participants had difficulty voicing their opinions, likely due to a language barrier.		
	English‐speaking participants	Non‐English‐speaking participants
Acceptance of Institutional Limitations	‘*I can't complain too much because the food has been reasonable’ (*A10) ‘*It's a hard thing that, to cater for everybody, you gotta be a bloody genius’* (A06) ‘*I understand, too, that that's difficult in the management side of things and providing all those things … having all these different cultures’* (A12)	‘*I don't complain, it's good enough’* (B04) ‘*I think they're doing their job. It's hard to please everyone. You can't please everyone’* (B08) ‘*I mean it's normal because too many people. But you (are) taking care of too many people. Is very good’* (B14)
There is Nothing to Complain About	‘*I'm happy with it all. Very good’* (A11) ‘*You eat what you can, leave what you can*’ (A05) ‘*If the table doesn't come across, I might just ask them to butter the bread for me’* (A09)	‘*I even wrote a note to them that I enjoyed the soup, thank you’* (B08) ‘*I'm extremely happy with the, with the, dietary services’* (B12) ‘*Sometimes I tell the, the people who are serving food to feed me’* (B10)
Clinical Care Is the Priority, Not Food	‘*I've got cancer everywhere in my body. It just seems immaterial to me that we're talking about food’ (A03) ‘No, I've I never talked to them about it. I don't think it's their worry. They've got enough looking after me medically, not for the food*.’ (A04)	‘*No, I don't, I don't talk all these things which they, they, they don't, they don't concern with the food or they feel concerned only with my illness and they never ask even*.’ (B07)
2. Food and Cultural Identity: Hospital food provided a stark reminder of cultural differences, with the non‐English‐speaking participants identifying themselves as the minority. They accepted that they should eat local food, but preferred one culturally familiar option per meal.		
Internalised Assimilation	N/A	‘*Cause they're not going to cook for me’ (B04)* ‘*You know us Asian’* (B05) ‘*For me all this, no, no … that's just too much work to do’* (B18)
Not Culturally Inclusive	N/A	‘*I cannot compare really what is the food they give it to me here’* (B15) ‘*Oh it's quite different, very different*’ (B08)
It's an Australian Institution	N/A	‘*Make [food] to more [like] at home’* (B02) ‘*Cannot criticise because they're serving at standard Australia’* (B04)
3. Experiences of Food Service: Multiple factors contributed to participants' overall experience of food service, with individual preference playing a key role in the outcome (positive or negative). Enablers of and barriers to food consumption became more evident.		
Taste, Temperature and Texture	‘*Taste has been good, yeah. Nice and fresh, you know’* (A12) ‘*Bland. No flavour’* (A06) ‘*By the time I get it, it's, the heats gone out of it. Still alright, it's warm*’ (A08)	‘*The food is very fresh and very yummy’* (B13) ‘*The taste is a little bit difficult in hospital’* (B09) ‘*Sometimes hard. I don't like it and then they take it back’* (B10) ‘*It's alright. Medium, not hold, not cold’* (B02)
Presentation and Usability	‘*I think they've been pretty exemplary, in there, you know presentation of it’* (A13) ‘*Umm you still have trouble getting the lid off, getting it, knives and forks ready … if you're in bed, you still have to manoeuvre, manoeuvre all the stuff and get it already*’ (A15)	‘*Everything is sort of so aesthetically, you know’* (B12) ‘*Plus a dish which is inside is too heavy, and if you're sick, you can't lift this heavy, heavy cover and take it*’ (B04)
Menu Options	‘*Yeah, I don't think I've had the same thing since I've been in here, so it's all good’* (A14) ‘*I haven't ordered any meal yet. When they bring it around, you got the sheet and it's marked general’* (A04)	‘*All times three choices or something like this. Each day, it's the choices’* (B18) ‘*Well, I prefer something else, but I don't know what they have because they don't give me a menu’* (B07)
Food Service Staff	‘*Some of them don't want to say anything but ahh others, you know they'll respond to a thank you or something. But others just say come in, present it and out they go’* (A02)	‘*Well, they're all polite, they all, they never rude or anything. And if I'm asleep, they will wake me up to eat my lunch or dinner’* (B07)
4. Nutrition and Health: An interplay existed between the hospital meal quality and participants' health, and participants' health impact on their food consumption. Overall, all participants ate small meal portions, believing their nutritional requirements were reduced in older age.		
Clinical Conditions and Impacts on Food and Nutrition	‘*I lost my taste altogether, ahh my mouth was very dry and I couldn't taste any food’* (A03) ‘*I think they're, they're quite nutritious the meals’* (A10) ‘*When you get good food, you don't have to be hungry’* (A08)	‘*Yesterday I was, a very bad day because my blood pressure was very, very high. I can't eat the dinner’* (B13) ‘*I need help because it's hard to carry up the food and the tool, I'll put it in my mouth. Because my hand shaking and I lose the food’* (B10) ‘*The dishes are especially considered in the diet, the dietary [nutritional] terms’* (B12)
Not Eating a Lot	‘*At my age, you know, I don't need the food and ahh so I umm I enjoy, I enjoy what I have, but as I say, I'm only a small eater’* (A02) ‘*The amount of food that I'm eating is very, very small … if I get through 25% of that [normal dinner plate], I'm doing real good’* (A05)	‘*I know because my mother, when you young, you eat more and when you older you eat less. So it was too much for me’* (B04) ‘*I could not even finish it. Was too much’* (B14) ‘*I just I can't swallow down the, the flavour I can't swallow it’* (B06)

#### 4.2.1 Theme 1: No Complaints Mindset

This theme captures the common mentality expressed by participants from both groups, who do not want to complain. There was an overarching mindset that complaining would not result in practical changes to the food service, with patients considering the complexities of the overall hospital system. The three sub‐themes explore why participants did not complain about their meals and the hospital food service.

##### 4.2.1.1 Acceptance of Institutional Limitations

Participants in both groups evaluated the food service and meals they received, acknowledging the large number of patients being served, and believed it was not feasible to cater to each individual. They did not want to be a burden, so they accepted that the meals might not suit their preferences. Participants explained that the hospital food was not bad, suggesting they had low expectations for the quality of the meals they would receive. ‘*I know the system, umm, you got a lot of people to feed, but it's different if you were just feeding me. But you know, for the amount of people they're doing, they're doing very well’* (A14).

NESB participants also shared similar sentiments:

You know it's not, it's not like restaurant, you're coming here enjoy. But it's it's good, good enough…

B14

##### 4.2.1.2 There Is Nothing to Complain About

The ‘no complaints’ mindset was also evident in the interviews, as many participants in both groups perceived the meals as adequate and meeting their expectations of hospital food. Most did not have any negative comments or constructive feedback to provide about the food service and were, instead, grateful for whatever they received. ‘*I feel the services are, are quite adequate. And umm I have no complaints’* (A02).

Instead of complaining, participants seemed to believe it was the hospital's role to provide the food and that it was their own responsibility to decide whether to eat their meals. ‘*But I have to eat, you know what they give it to me, you know’.*

B05

If participants were to discuss the food service with staff, they would either request a minor adjustment to their meal, seek help with meal preparation, or offer general feedback. ‘*I've often said, “Could I have?” and they go back to the kitchen and find it and bring it to me’.*

A04

These types of requests were more common in the English‐speaking group, with only one NESB participant sharing that they had asked for help from staff.

##### 4.2.1.3 Clinical Care Is the Priority, Not Food

Many English‐speaking participants did not complain about their food because it was a lower priority than their health. They preferred that staff focus on their illnesses and healthcare rather than on the food service. ‘*I've got cancer everywhere in my body. It just seems immaterial to me that we're talking about food’* (A03). Another participant stated: ‘*No, I've I never talked to them about it. I don't think it's their worry. They've got enough looking after me medically, not for the food’* (A04).

A similar sentiment was apparent among NESB participants who felt it was appropriate that clinical staff were focused on their illness rather than their mealtime satisfaction.

No, I don't, I don't talk all these things which they, they, they don't, they don't concern with the food or they feel concerned only with my illness and they never ask even.

B07

#### 4.2.2 Theme 2: Food and Cultural Identity

The NESB participants perceived themselves as separate from other English‐speaking patients, with the lack of culturally familiar food contributing to a feeling of being the minority.

##### 4.2.2.1 Internalised Assimilation

In the interviews, NESB patients referred to themselves as the minority and seemed to believe they should conform to the meals provided to them.

Because I'm the only one and this is a big organisation, so I think it's okay. For one person, it is not alright to just ask them.

B15

Multiple NESB participants reported that family members provided them with food, as they were dissatisfied with the hospital meals. However, the English‐speaking group mentioned how their families brought snack foods, such as Vegemite or biscuits, in addition to the hospital meals.

Granddaughter sneaked, a packet of biscuits and she asked her nanny what sort of biscuits I like and she, she said he likes the ginger nuts.

A16

##### 4.2.2.2 Not Culturally Inclusive

The NESB participants did not view the hospital meals as culturally inclusive. They felt that the meals did not compare to the types of food they were used to eating at home. One participant stated, ‘*It might be alright, I'm just not used to eating that kind of food’* (B06), and others shared sentiments such as ‘*Actually it is good. But the problem is that I am not used to of it’* or ‘*Oh, it's quite different. Very different’ (B08).*

Multiple NESB participants preferred foods originating from their cultural background, including an Italian‐born participant who requested ‘*a little bit of pasta’* (B02) and a patient born in the Philippines who stated,

I prefer if they give me some noodles, but they don't have any noodles.

B05

##### 4.2.2.3 It's an Australian Institution

This sub‐theme reflected the understanding of NESB participants that the hospital serves ‘Australian food’ to Australian standards, including meal type and timing. Participants did not want the whole menu changed; they preferred an additional option at each meal to be more culturally appropriate.

Not too much because Australian institutions, but have options for migrants as well.

B04

One NESB participant clearly articulated the need to cater to cultural inclusivity.

I think because they, they are cooking it would be nice, just have one option which is coming from different country (unclear) 1 day, 1 days of Chinese, 1 day Polish, 1 day some Indian or something because, because there's plenty of people here, not born in Australia.

B04

Interestingly, this sentiment was also shared by English‐speaking participants.

I suppose the hardest part for these people working here, in the catering, how do you cater for, I don't know how many people are in this hospital, say there's a thousand people, thousand meals and you might have thirty different nationalities. How do you cater for all nationalities food? It's very difficult.

A05

#### 4.2.3 Theme 3: Experiences of Food Service

For all participants, the menu, meal quality and staff were integral parts of the hospital food service experience. These aspects were mentioned in all the interviews, but their individual preferences influenced how they were experienced, either positively or negatively.

##### 4.2.3.1 Taste, Texture and Temperature

There were mixed experiences with the flavour of the hospital meals within and between the two groups. Some participants from both groups stated that they were satisfied, although this could be attributed to the ‘No Complaints Mindset’ *(Theme 1)*, as illustrated by the following comment, which positions food refusal and dissatisfaction as an intrinsic factor.

Well, I've got no complaints about it. Just what I've had and I haven't had a lot. Umm I have nothing to complain about. Even if I sent the meals back, it's because of me because I couldn't eat it.

A07

In contrast, other English‐speaking participants found the food bland, and some NESB participants disliked the flavour.

Oh [the] hospital food? One word, bland’ (A05) and ‘[I] don't like the taste.

B07

Regarding ‘texture’, a few participants mentioned that their food was either too dry or too hard, making it difficult to eat.

The Mashed potato tended to be a little bit dry and you know ahh but ahh and some of the vegetables, there was some carrot and some brussels sprouts I had the other day were a bit, bit hard.

A12

Participants' experiences with the meal temperature varied; some reported it was cold or warm, while others said it was just right. Comments such as this were common across both groups.

Yeah, it's never, I wouldn't say never, but it's on the, on the tepid side mostly. I've never had a food that's been stone cold and inedible. But it's generally on the, on the warm side of things.

A13

##### 4.2.3.2 Presentation and Usability

Overall, participants across both groups reported that they liked the presentation of their meals and found the appearance acceptable.

Ahh positively, but mainly all looked good when it came. You know lift the lid, it all looks alright.

A14

However, there were mixed responses regarding the eating experience and the usability of the provided serving ware. Some participants had difficulty cutting the meals themselves, while others struggled to open containers and lids on dishes as they were too heavy. Participants across both groups shared similar sentiments ‘*Ahh the way that it comes in it's containers and some of them are a bit hard to open’* (A04) and ‘*For me only, as you said container or there serving lunch or, or dinner, to me is too, too heavy’* (B04). For patients who did not request assistance with setting up their meals, it is possible that family visitors were able to help.

I'll be alright, my daughter help[s] me.

B02

##### 4.2.3.3 Menu Options

Most participants were happy with the number of options offered on the hospital menu. The ability to choose between options was a strong preference for participants, increasing the likelihood that they would have a meal they wanted to eat.

Because the ones I don't like, there's enough of the other ones I like that can cover that anyway.

A16

However, one longer‐stay patient (A05, 10 weeks) explained that their meals had become too repetitive. Additionally, four English‐speaking and one NESB participant shared that they had not been given the option to choose their meals and were provided with standard menu options.

##### 4.2.3.4 Food Service Staff

Participants shared their experiences with the hospital food service staff, who were generally described as polite when delivering their meals. However, some staff would make an extra effort to provide more person‐centred care by having a brief conversation or ensuring patients could reach their meals.

The people are different … when they see that you are very sick, [some] try to help you … some people just leaving and going.

B04

English‐speaking participants spoke more about interactions with the food service staff or described their interactions*. ‘We usually have a two‐ or three‐seconds conversation of what was the food like today or you know, there was a little bit of person to personal interaction*’ (A05).

#### 4.2.4 Theme 4: Nutrition and Health

Theme 4 captured the relationship between participants' health and the quality of hospital meals, describing their experiences with consuming small meal portions.

##### 4.2.4.1 Clinical Conditions and Impacts on Food and Nutrition

Participants' health conditions and age‐related factors presented a barrier to consuming and enjoying the hospital meals. For example, some participants modified their meal choices to align with their therapeutic needs; ‘*I don't have a soup because I'm on a liquid restriction diet’* (A06). Alternatively, some participants experienced clinical symptoms, such as changes in taste, that affected their enjoyment of meals. As such, food was not just about providing patients with nutrition to support their health; the meals were also valued for enjoyment.

Even when you are in hospital, you are sick, you not only eat to be alive, but eat to have some pleasure.

B04

##### 4.2.4.2 Not Eating a Lot

It was common for participants to eat small portions of their meals due to low appetite or dissatisfaction with the food served. One NESB participant stated: ‘*I never even eat all my meal. It's, I order only small one’* (B05). For some participants, the combination of diminished appetite and meal dissatisfaction led to refusing the meal entirely.

Ohhh over the last four weeks I've been in here, it would have been a few times, I'd take the lid off and I look at it and I'd go ughh and put the lid back

A10

Participants considered the hospital meals to be nutritionally adequate to meet their needs, which they believed were reduced due to being older people with illnesses engaging in low activity levels. More than half of the participants expressed this perception of reduced nutritional needs in older age, most directly mentioning a preference for smaller‐sized meals.

I sort of say I'll just have a small serve and that's sufficient, you know, cause I'm not doing a lot of exercise or anything.

A12

If served larger meals, some participants were unable to eat them all, with one noting that the larger portion was unappealing.

When they bring me the big size, that was putting me off a bit, I couldn't manage all that so.

A07

## 5 Discussion

To our knowledge, this study was the first to investigate the lived experience of hospital food service for older patients in relation to their cultural and linguistic backgrounds, using in‐depth qualitative interviews. To date, quantitative research suggests that NESB patients with low food service satisfaction have an increased likelihood of complications and delayed discharge compared to patients from English‐speaking backgrounds [14], but provides limited insight into the potential reasons why. In the present study, we found distinct differences among the NESB participants' experiences of the hospital food service relating to the lost connection between food and their cultural and linguistic background. Language barriers also hinder communication with food service staff and pose challenges to their ability to consume food. However, the two groups shared a ‘no complaints’ mentality and preferred smaller meals.

### 5.1 Findings Related to Cultural Familiarity

For both English‐speaking and NESB patients, food and culture emerged as a central theme. Food and mealtimes are well‐recognised as ways to maintain cultural heritage and identity, as shown in qualitative interviews with Indian, Chinese and Malay women in Singapore [22]. In our study, however, NESB participants described limited cultural inclusivity in hospital food services and a lack of connection with the meals provided. They often referred to themselves as a minority and felt obliged to conform to the ‘Australian’ meals on offer. Similar findings were reported in a qualitative study of older migrants with dementia, who experienced cultural loss through unfamiliar food and drink, despite clinical needs taking precedence [23]. Unfamiliar meals have also been associated with lower sensory ratings for appearance, aroma, taste and texture [24], suggesting that the absence of culturally familiar foods may reduce both enjoyment and willingness to eat. Other research with ethnic minority patients likewise found that hospital food, while accepted, was markedly different from what they were accustomed to consuming and did not align with their mealtime enjoyment [25].

The lack of culturally familiar food provided within the hospital presents a barrier to food consumption for the NESB patients interviewed. This finding is consistent with our 2019 survey of public hospitals in New South Wales, which revealed that patients from NESB experienced more unfavourable outcomes, including dissatisfaction with hospital care and delayed discharge due to feeling unwell [14]. This may be explained, at least in part, by low food intake due to a lack of suitable food options. Participants expressed that they did not want the entire hospital menu changed but preferred a more culturally appropriate and familiar option to be available on a regular basis. Similar preferences were identified in a study of patient mealtime experiences, with desired improvements to hospital food quality, including the increased availability of menu options that cater to patients' cultural needs [26].

### 5.2 Findings Related to Languages and Communication Styles

Cultural differences in the hospital food service also extended to language and communication barriers for the NESB participants. When patients cannot communicate with health professionals, including food service staff, in their own language, it becomes challenging to gather their feedback and tailor care to meet their individual needs and preferences [23]. Low language proficiency can inhibit effective communication, reducing the desire of older patients from minority backgrounds to communicate or ask questions [27]. This passive participation in healthcare [23, 27] is consistent with our findings, as NESB participants reported that they would not inform staff if they disliked the food, instead leaving their meals uneaten. English language barriers may also have prevented NESB participants from asking staff for help with meal setup, which is problematic, as it is known that not receiving assistance when needed is a common barrier to food intake in hospitals [26, 28].

### 5.3 Findings Related to a No‐Complaint Mindset

An overarching similarity between the English and NESB groups was their ‘no complaints’ mindset. This mentality is reflected in a scoping review of older patients' perspectives on hospital mealtimes, in which they believed they should not complain or burden staff and instead should eat and be satisfied with the meals they are served [29]. This common characteristic of the Silent Generation, born between 1928 and 1945, aligns with the median demographic age of our participants. This population group tolerates personal discomfort, wanting to conform and reduce their burden on others [30]. This attitude presents a significant barrier for older patients to receive quality meals and food services that align with their preferences, as they are unlikely to speak up for themselves and their needs. Similarly, our participants were also aware of the catering requirements within the larger hospital system and adjusted their expectations accordingly, often describing meals as ‘not bad’ or ‘good enough’. The expectation‐disconfirmation paradigm may explain this viewpoint; if participants had low expectations for the hospital food, they would experience positive disconfirmation if the meals were better than expected [31]. To some extent, pre‐empting poor meal quality and participants' ‘no complaints’ mindset may have alleviated dissatisfaction. This is not uncommon in the experiences of older patients with hospital mealtimes, characterised by reduced expectations regarding menu choice [29] and the availability of staff assistance during mealtimes due to high workloads [32]. However, our study presented a few examples in which food service staff supported participants' intake by providing patient‐centred care, including ensuring their meals were set up. Mealtime assistance from staff or volunteers, such as opening packages, cutting food and physically feeding patients, has been shown to increase food intake in older patients in hospital and rehabilitation settings [33]. This is possible using robotics in the near future [34] to solve the issue and make mealtime assistance more comprehensive and tailored to patients' needs.

### 5.4 Findings Related to Portion Sizes

Both groups also preferred small meal portions, with a predominant belief that their nutritional requirements were reduced with age. Participants often alluded to not completing their meals due to health constraints, with these physiological or pathological changes likely affecting their nutrition [35]. Medical conditions and age‐related factors are common barriers to food intake and impact our participants' ability and desire to eat [26]. For example, illness was the most common barrier to food intake experienced by adult patients in acute care across Canada [28]. Considering older people's reduced food intake and potential loss of appetite associated with clinical conditions [36], it is even more important to meet patients' tastes and cultural preferences to increase overall consumption. Although all participants preferred smaller meals, providing food that met their individual preferences, particularly in terms of taste, appeared to facilitate their intake. The personalisation of hospital meals, tailored to patient choice and fluctuations in appetite, has previously been identified as a means of improving food intake [37].

### 5.5 Strengths and Limitations

To our knowledge, this is the first study to report on the lived experience of hospital food services among older patients from English‐speaking and non‐English‐speaking backgrounds, aiming to understand any gaps in quality of care and equity in accessing care. The use of semi‐structured interviews and open‐ended questions enabled participants to share more personal and in‐depth experiences, thereby addressing the research aim. However, convenience sampling resulted in limited diversity among the NESB participants, who were primarily born in several European countries, potentially limiting the generalisability of our findings to other NESB cultures in the East [38]. Similarly, patients' experiences were limited to one hospital food service system. A potential bias exists due to participants' ‘no complaints’ mindset, which is often observed among older patients [30]. This may have led to under‐reporting of dissatisfaction with the hospital food service or more neutral responses. Furthermore, the interview length was significantly shorter for NESB participants, who provided less detailed responses despite the use of a translator, possibly due to communication barriers associated with using translation services over the telephone. As such, we recommend in‐person translation services for older adults in similar research to facilitate qualitative interviews, thereby maximising the length and quality of the interviews. Additionally, we did not ask participants about the length of stay in Australia as we felt it could be perceived as a private, sensitive question. As the hospital was public and given the participants' advanced age, we assumed that they had been living in the country for many years. The imbalance of gender distribution between the groups may affect the responses and lived experience of the groups. Despite significant efforts to balance the gender distribution, we adopted a convenience sample approach, as the patients were older and nearing discharge, necessitating a prompt recruitment strategy.

### 5.6 Implications and Potential Solutions

#### 5.6.1 Offering More Culturally Familiar Meal Options

The lack of culturally familiar food emerged as a barrier to meal satisfaction, contributing to a cultural disconnect for people from NESBs, who often relied on family members to provide familiar food. Addressing this issue by implementing practical menu modifications [39], such as having culturally familiar snacks or menu staples like rice, Lebanese bread or pasta, may help accommodate a broader range of patients' needs. Additionally, offering at least one culturally appropriate option per menu cycle may increase food intake and enhance the dining experience for NESB patients [40].

#### 5.6.2 Increased Availability of In‐Person Translation Services

Language barriers persisted despite the use of translation services in interviews with NESB participants, limiting their ability to express themselves effectively. Therefore, the advancement of translation services [41], particularly those using artificial intelligence [42], may offer cost‐effectiveness in fostering patient–staff communication for patients with limited English proficiency [43].

#### 5.6.3 Fostering Open Communications Between Patients and Healthcare Professionals Regarding Hospital Food

Both groups shared a ‘no complaints’ mentality, often leading to a poorer patient meal experience and unmet needs. Staff training focused on proactive strategies, such as hospital staff initiating conversations about meal satisfaction, may help patients express their individual needs and preferences without voicing complaints and counteract this mindset. Additionally, patient‐centred care initiatives such as systematic patient engagement should be implemented to identify and address barriers to adequate food intake and improve patient satisfaction [44]. Patient feedback on meal options was found to be a critical tool for enhancing cultural inclusivity in dietary services [40].

## 6 Conclusions

This qualitative study of 30 older patients in a public hospital in Australia highlights the intersection of hospital food services with cultural identity, communication and patient care. Findings demonstrate the importance of offering culturally familiar food options to better meet the needs of patients from diverse backgrounds, as well as strengthening patient feedback mechanisms and staff–patient communication to ensure that meals align with individual dietary needs. By identifying enablers and barriers to meeting cultural and nutritional requirements, this study advances understanding of how hospital food services can promote equity, diversity and inclusion. This study adds to the limited body of evidence on the patient experience of hospital food, particularly among culturally and linguistically diverse populations. It underscores the broader role of food in enhancing both nutritional outcomes and the overall quality and inclusiveness of healthcare delivery.

Future research would be beneficial in investigating the long‐term effects of implementing culturally inclusive food practices in hospital food services on patient satisfaction and recovery outcomes. Comparative studies involving different cultural and ethnic groups and age cohorts could also provide a better understanding of how cultural and generational differences shape patient experiences in hospital food services.

## Author Contributions

Conceptualisation and design of the study: Zhaoli Dai and Yogesh Shama. Methods: Zhaoli Dai, Morgan Pankhurst and Caitlin Wyman. Data collection: Sue Yi Lee and Caitlin Wyman. Data analysis and synthesis: Caitlin Wyman, Morgan Pankhurst and Zhaoli Dai. Results interpretation: all authors. First draft of the manuscript: Caitlin Wyman. Review and revision of the manuscript: all authors. Funding and resources: Zhaoli Dai and Yogesh Shama.

## Conflicts of Interest

The authors declare no conflicts of interest.

## Supporting information

Supp_File_1_submitted.

**Supplementary File 2:** Interview guide.

**Supplementary File 3:** Details of participant characteristics.

## Data Availability

The data that support the findings of this study are available from the corresponding author upon reasonable request.
