# Supplementary material for: Hospital Food Service Experiences Between Older Patients From English‐ and Non‐English Speaking Backgrounds in a Large Public Hospital in Australia: A Qualitative Analysis
Source: Health Expect. 2025 Sep 26;28(5):e70444. doi: 10.1111/hex.70444 (PMC12465006; doi:10.1111/hex.70444)
Supplement: Supplementary file 2 — Interview guide. [file HEX-28-e70444-s001.docx]

**Supplementary File 2. Interview guide**

**Interview themes for hospital meal experiences:**

• Can you start with your overall experience with the hospital food services at Flinders Medical Centre?

• Can you think about the quality of the food and tell me your preferences?

• How would you describe whether the meals meet your dietary needs?

**•** How would you describe how the meals meet your dietary needs according to your culture and religion?

**•** In your opinion, what needs to be changed in this hospital so that you will eat the food served in the hospital to meet your cultural and ethnic background?

• If you can change the meals to suit your cultural and ethnic background, what would you like to change?

• What do you think of the nutritional values of the hospital meals?

**•** Can you comment on food packaging, cutlery, hygiene, and food waste? What do you think of any of these aspects?

• Now, can you describe the quality of the hospital food services?

**•** If you can change how hospital meals are served, what aspects do you want to change? What are the best and worst aspects of the meal service?

**•** Do you need support to eat at mealtime?

• Have you had an opportunity to talk about the food quality and hospital food service with a doctor, nurse, and/or dietician? What did you tell them, and how did they respond? After that, did things improve in terms of how you consumed your meals, and were the food and services enhanced?

• How much longer will you stay in the hospital?
