# Supplementary material for: Hospital Food Service Experiences Between Older Patients From English‐ and Non‐English Speaking Backgrounds in a Large Public Hospital in Australia: A Qualitative Analysis
Source: Health Expect. 2025 Sep 26;28(5):e70444. doi: 10.1111/hex.70444 (PMC12465006; doi:10.1111/hex.70444)
Supplement: Supplementary file 3 — Details of participant characteristics. [file HEX-28-e70444-s003.docx]

**Supplementary File 3. Details of participant characteristics**

| **Participant** | **First language** | **Country of birth** | **Interview length** | **Use of translation services** | **Length of stay (days)** | **Admission reason** |
| --- | --- | --- | --- | --- | --- | --- |
| A02 | English | UK/England | 20m39s | No | 5 | Fall, broken ribs |
| A03 | English | Australia | 20m45s | No | 21 | Cancer |
| A04 | English | Australia | 27m22s | No | 6 | Infections, inconclusive |
| A05 | English | Australia | 31m24s | No | 70 | Back pain |
| A06 | English | UK/England | 24m04s | No | 7 | Respiratory, difficulty walking |
| A07 | English | Australia | 23m09s | No | 14 | Gastrointestinal |
| A08 | English | Australia | 24m57s | No | 5 | Enema |
| A09 | English | Australia | 25m42s | No | 14 | Infection, epistaxis |
| A10 | English | Australia | 24m43s | No | 28 | Pulmonary oedema |
| A11 | English | Australia | 19m19s | No | 60 | Unknown |
| A12 | English | Australia | 25m58s | No | 4 | Dizziness, shortness of breath |
| A13 | English | Australia | 22m33s | No | 3 | Multiple myeloma, Parkinson's, blood pressure, cardiac |
| A14 | English | Australia | 15m43s | No | 5 | Heart failure |
| A15 | English | Australia | 21m15s | No | 3 | Fall |
| A16 | English | Australia | 17m06s | No | 7 | Rheumatoid arthritis |
| B02 | Italian | Italy | 16m50s | Yes | 4 | Infection |
| B04 | Polish | Poland | 24m14s | No | 21 | Back pain, bacterial infection |
| B05 | Filipino | Philippines | 23m31s | No | 21 | Hyperhidrosis, swollen legs |
| B06 | Greek | Greece | 10m34s | Yes | 3 | Hypertension, dizziness |
| B07 | Greek | Egypt | 11m59s | No | 7 | Fall |
| B08 | Hindi | Fiji | 11m40s | No | 21 | Heart attack, kidney failure, pulmonary oedema |
| B09 | Dutch | Netherlands | 7m46s | No | 5 | Fall |
| B10 | Kinyarwanda | Congo | 21m15s | Yes | 28 | Fall with broken bones |
| B12 | Polish | Poland | 28m29s | Yes | 14 | Stroke |
| B13 | Polish | Poland | 18m21s | No | 7 | Respiratory, difficulty walking |
| B14 | Croatian | Croatia | 7m08s | No | 4 | Cardiac issues |
| B15 | Punjabi | India | 13m57s | No | 21 | Infection |
| B16 | Serbian | Croatia | 11m10s | Yes | 10 | Swollen leg |
| B17 | Polish | Poland | 7m | No | 6 | Respiratory |
| B18 | German | Germany | 8m31s | No | 6 | Hip replacement, lymphoedema |

1. Represents participants born in English-speaking countries (Australia or the United Kingdom).
2. Represents participants from non-English speaking backgrounds.
